# Low-iodine 40-keV virtual monoenergetic CT angiography of the lower extremities

**DOI:** 10.3389/fcvm.2023.1276738

**Published:** 2023-10-23

**Authors:** Guillaume Fahrni, Thomas Mingas, Arthur Deliessche, Smail Hraichi, David C. Rotzinger, Salim A. Si-Mohamed, Sara Boccalini, Philippe Douek

**Affiliations:** ^1^ Department of Diagnostic and Interventional Radiology, Cardiothoracic and Vascular Division, Lausanne University Hospital and University of Lausanne, Lausanne, Switzerland; ^2^ University Lyon, INSA-Lyon, University Claude Bernard Lyon 1, UJM-Saint Etienne, CNRS, Inserm, CREATIS UMR 5220, U1206, F-69621, Villeurbanne, France; ^3^ Department of Radiology, Hôpital Louis Pradel, Hospices Civils de Lyon, Bron, France

**Keywords:** DECT, CT angiography, lower extremities, 40-keV, monoenergetic, peripheral artery disease, reduced iodine volume, arteries

## Abstract

**Introduction:**

To evaluate a reduced iodine volume protocol for lower extremity CT angiography (CTA) using dual-energy CT (DECT).

**Methods:**

This retrospective study included consecutive patients who underwent lower extremity CTA from June to December 2022. A 10 ml 1:1 mixed test bolus was performed, followed by a 40 ml full bolus at a 2.5/s injection rate, using 400 mg/ml iodine contrast media. Conventional and 40 keV virtual monoenergetic images (VMI) were reconstructed. For both reconstructions, five main artery segments were assessed with a 3-point image quality score as well as quantitative attenuation, signal-to-noise ratio (SNR) and contrast-to-noise ratio (CNR) measurements with diagnostic quality thresholds (respectively >150 HU and >3).

**Results:**

Forty patients were included in the study (mean age 68 ± 12 yo). 200 artery segments were assessed. Median qualitative image scores were 3 [IQR, 3, 3] for both reconstructions. 40 keV VMI upgraded qualitative scores for 51 (26%) of patients, including 9 (5%) from nondiagnostic to diagnostic quality. 40 keV VMI obtained attenuation and CNR diagnostic quality for respectively 100% and 100% of segments, compared with 96% and 98% for conventional images (*p* < 0.001). Distal artery segments showed the most differences between 40 keV VMI and conventional images.

**Conclusion:**

A low-iodine lower extremity CTA protocol is feasible, with 40 keV virtual monoenergetic spectral reconstruction enabling maintained diagnostic image quality at the distal artery segments.

## 1. Introduction

Peripheral artery disease (PAD) of the lower extremities is the third leading cause of atherosclerotic vascular morbidity after coronary heart disease and stroke (1). Atherosclerosis plaque formation leads to lower limb ischemia, causing intermittent claudication that can evolve to rest pain, ulceration and gangrene (2). It is linked with traditional cardiovascular disease risk factors such as smoking, advanced age, diabetes, hyperlipidemia, hypertension and hypercholesterolemia (3). The diagnosis is based on history, symptoms, clinical examination, measurement of the ankle-brachial index, toe-brachial index and imaging (4). Routine screening for PAD is reserved for individuals with numerous risk factors. Historically, invasive digital subtraction angiography was the gold standard diagnostic method but is now reserved for treatment, due to the risks of complications (5).

Current diagnosis imaging modalities include duplex ultrasonography (DUS), computed tomography angiography (CTA) and magnetic resonance angiography (MRA) (6). CTA has the advantages of high spatial resolution, arterial wall and plaque characterization as well as fast image acquisition (7). CTA has a sensitivity of 95% and a specificity of 96% for identifying hemodynamically significant lesions (8). Drawbacks of CTA include radiation exposure and the need to use iodinated contrast media that can cause contrast-induced acute kidney injury (CI-AKI) (9). This is problematic, as lower extremity CTA is often performed for elderly patients with other AKI risk factors such as diabetes and hypertension. Iodinated contrast media is particularly nephrotoxic in patients with a glomerular filtration rate (eGFR) less than 30 ml/min/1.73 m^2^ (10). Moreover, an effort should be made to reduce iodine usage due to recent concerns regarding the ecological impacts of iodinated contrast media in drinking and surface water (11) and the risks of contrast media shortages, such as during the covid-19 pandemic (12). In the current guidelines, high-iodine (350–400 mg/ml) contrast media are used (13, 14), with a volume of 120 to 140 ml (4, 7), with some reduced contrast volume protocols using down to 80 ml (15). Lower iodine volumes protocols such as at 45–50 ml have been successfully performed for aortic and lower extremity CTA (16–20) but not yet at 40 ml.

Recent CT technological advances led to the introduction of dual-energy CT (DECT) that offers better tissue characterization due to CT attenuation values obtained at two different x-ray energy levels, either by applying two different tube potentials (usually 80 and 140 kVp) (21), or by discriminating two energy levels at the detector side (22). This enables the possibility of reconstructing virtual monoenergetic images (VMI) at discrete energy levels down to 40 keV. Using low keV VMI enhances iodinated contrast in the image, enabling iodine dose reduction. Multiple studies have recently shown the interest of using 40 keV VMI to improve diagnostic accuracy and image quality in different vascular settings and with different CT detector technologies such as dual source, dual layer and fast kVp switching (23–25). In a phantom study, up to 40%–60% iodine reduction could be achieved without loss in image quality (26). Similar results were obtained in-vivo with coronary, pulmonary, and aortic CTAs (27–31).

Therefore, we aimed to evaluate the subjective and objective quality of a reduced contrast volume DECT of the lower extremities protocol, with a bolus of 40 ml of iodinated contrast media, using conventional and 40 keV VMI.

## 2. Materials and methods

### 2.1. Study design and population

In this monocentric, retrospective study, patients referred for lower extremity DECT were included from June to December 2022. Informed consent was waived due to the retrospective nature of the study. Exclusion criteria were age < 18 years, iodinated contrast media allergy, and renal insufﬁciency with an eGFR less than 30 ml/min/1.73 m^2^.

### 2.2. DECT scanning protocol

**Figure 1:**
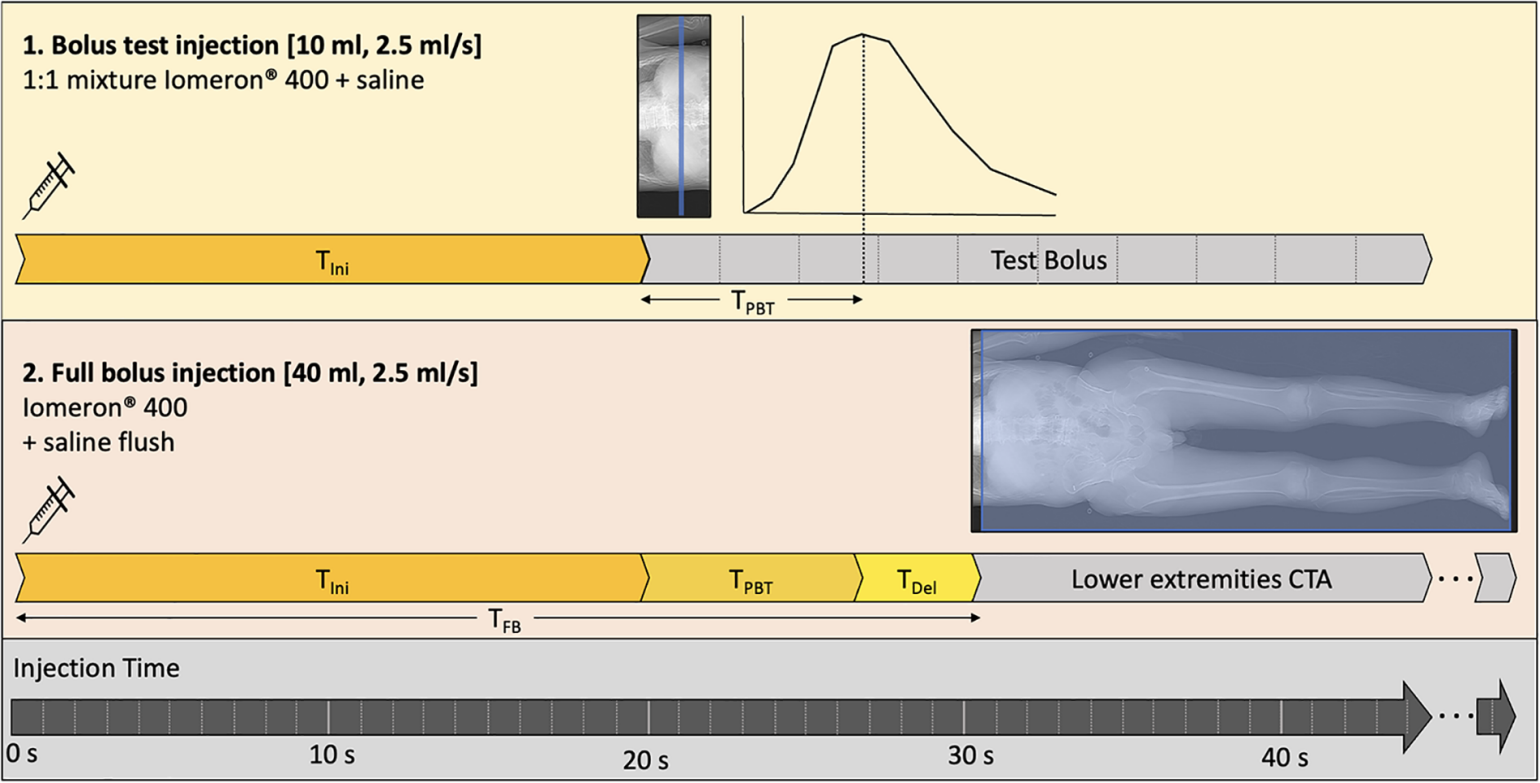
Injection protocol. First, a 10 ml diluted test bolus is performed after a 20-sec initial time after injection start (*T*_Ini_) to assess the peak test bolus time (*T*_PTB_). Then, the full 40 ml bolus is injected, and image acquisition start at the full bolus time (*T*_FB_) equal to the sum of *T*_Ini_ and *T*_PTB_ with an added delay time (T_Del_) of 4 s.

All examinations were performed on an 8-cm collimation second-generation dual-layer 7,500 CT system (Philips Healthcare). The acquisition and reconstruction parameters are summarized in Table 1. The injection protocol was performed with a power injector (Bracco), using Iomeprol (400 mg/ml; Iomeron®, Bracco), through an 18–20G catheter inserted into an antecubital vein. First, a test bolus, which is one of the recommended techniques (32), was performed on the infrarenal abdominal aorta with a 10 ml 1:1 mixture of contrast media and saline solution, with an injection rate of 2.5 ml/sec, followed by a 30 ml saline flush. Test bolus recording consisted of 10 consecutive slices at 2.5 s intervals, starting with an initial time (*T*_Ini_) of 20 s after injection. The test bolus peak time (*T*_BTP_) was manually measured. An empirically chosen 4 s delay time (*T*_Del_) was added to avoid outrunning the bolus due to table speed. The resulting full bolus time (*T*_FB_) was thus defined as: *T*_FB = _*T*_Ini + _*T*_BTP + _*T*_Del_, which corresponded to an average 30.4 ± 5.6 s depending on the patient. Finally, the full bolus of 40 ml of non-diluted contrast media was injected, and image acquisition started at T = *T*_FB_ at a rate of 2.5 ml/sec, followed by a 20 ml saline flush. Images were acquired from the diaphragm to the lower extremities. The injection protocol is summarized in Figure 1. Finally, conventional images and 40 keV VMI were reconstructed for each patient.

**Table 1:** Acquisition and reconstruction parameters.

Parameter	Value
Tube voltage (kVp)	100 kVp
Tube current (mAs)	89 mAs
Dose modulation	Automated dose modulation with dose right index of 14
Rotation time (s/rot)	0.75
Pitch	0.63
Collimation (mm)	128 × 0.625
FOV (mm)	300
Matrix size (pixels)	512 × 512
Slice thickness (mm)	1
Slice increment (mm)	0.6
Reconstruction kernel	B
Iterative reconstruction	Iterative model reconstruction level 1

FOV, field of view.

### 2.3. Qualitative analysis

Three radiologic technologists (SH, TM and AD) with 8, 5 and 2 years of experience qualitatively evaluated the image quality by consensus using a 3-point scale (1: nondiagnostic, 2: suboptimal, 3: optimal). A radiologist (GF) with 4 years of experience in vascular imaging performed the same evaluation, blinded to the other groups results. Images were reviewed on a dedicated clinical workstation (Intellispace Portal, Philips Healthcare), using 2D viewing mode in axial plane. Zooming and window adjustments were allowed. Arterial segments of different sizes were assessed along the vascular tree of the lower extremities. The abdominal aorta, external iliac artery, superficial femoral artery, popliteal artery, and anterior tibial artery were chosen for evaluation. Only the arteries on the right side were assessed. As image quality in segments with total occlusion is not contrast-dependent, they were excluded, and collateral pathways were assessed instead. The number of upgraded scores and saved exams (upgrade from a nondiagnostic score of 1 to a diagnostic score of 2 or more) were assessed.

### 2.4. Quantitative analysis

Three radiologic technologists (TM, AD and SH) performed attenuation measurements on conventional images and 40 keV VMI. A circular region of interest (ROI) was manually placed on each of the abovementioned arterial segments. The ROI was drawn from the center of the vessel, with a min. surface of 5 mm^2^, expanding as widely as possible within the lumen, without contact with the arterial wall or calcifications/atherosclerosis plaque. Mean Hounsfield units (HU) and noise—i.e., standard deviation—were assessed. A CT number of 150 HU was used as a cutoff for diagnostically adequate opacification (33).

A radiologist (GF) measured background attenuation with ROIs placed in the quadriceps muscle tissue, with a min. surface of 10 mm^2^, avoiding fatty streaks and density artifacts near bones. Background mean HU and noise were assessed. For each arterial segment, the signal-to-noise ratio (SNR) and contrast-to-noise ratio (CNR) were calculated for both image modalities using the following formulas (30):

$$\mathrm{SNR}\;=\;\frac{\mathrm{meanH}U_{\mathrm{artery}}}{SD_{\mathrm{muscle}}}$$

$$\mathrm{CNR}\;=\;\frac{\left|\mathrm{meanH}U_{\mathrm{artery}}-\mathrm{meanH}U_{\mathrm{muscle}}\right|}{\sqrt{\frac{1}{2}({\mathrm{SD}}_{\mathrm{artery}}^{2}+\;{\mathrm{SD}}_{\mathrm{muscle}}^{2})}}$$

Image quality was considered diagnostically adequate for a CNR > 3.0 (34). To avoid any discrepancies, all ROIs were first drawn on conventional images and then copied and pasted to the exact same position on the 40 keV VMI.

### 2.5. Statistical analysis

Statistical analysis was conducted using SPSS Statistics version 26.0 (SPSS Inc., Chicago, IL, USA). Normality of data distribution was assessed using a Kolmogorov–Smirnov test. Continuous variables are expressed as mean ± SD. Ordinal variables are expressed as median [IQR], unless otherwise specified. For qualitative analysis, inter-reader agreement was quantified using weighted kappa coefficients, and defined as follows: ≤0, poor; 0.01–0.20, slight; 0.21–0.40, fair; 0.41–0.60, moderate; 0.61–0.80, substantial; ≥0.81, excellent. Differences between median quality scores were evaluated with the Mann-Whitney U test. For quantitative analysis, attenuation, SNR and CNR differences between conventional images and 40 keV VMI were evaluated with a Wilcoxon two-sample test. A *p* value < 0.05 was considered statistically signiﬁcant.

## 3. Results

### 3.1. Population characteristics and scanning protocol

A total of 40 patients were included [mean age 68 ± 12 yo, 32 men (80%)], resulting in a total of 200 artery segments. Patients' characteristics and risk factors are detailed in Table 2. Mean body mass index (BMI) was 26.3 ± 4. 4 (93%) patients presented risk factors for PAD, the most prevalent risk factor was smoking, affecting 31 (78%) patients.

**Table 2:** Patient characteristics.

Parameter	Value
Patients (*n*)	40 (100%)
Men (*n*)	32 (80%)
Women	8 (20%)
Age (yo)	68 ± 12
Weight (kg)	78 ± 13
Height (cm)	170 ± 9
BMI (kg/m^2^)	26.3 ± 4.1
Risk factors	*n* (%)
Smoking	31 (78%)
Type II diabetes	8 (20%)
Hypertension	27 (68%)
Dyslipidemia	24 (60%)
Obesity (BMI ≥ 30 kg/m^2^)	10 (25%)
Age > 65 yo	24 (60%)
Family history of PAD	17 (43%)

BMI, body mass index; PAD, peripheral artery disease. Data expressed as total (percentage) or mean ± SD.

### 3.2. Qualitative image quality

**Figure 2:**
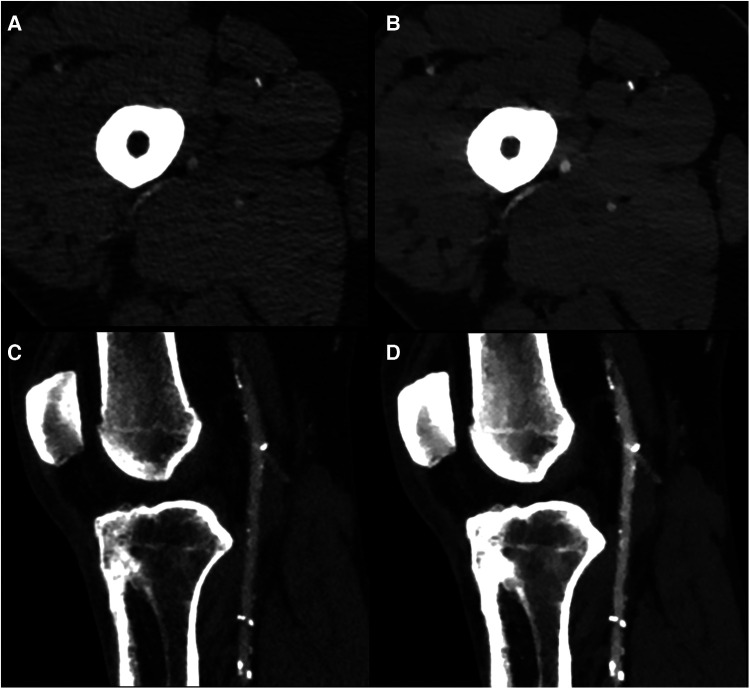
Example of conventional images (**A,C**) and 40 keV VMI (**B,D**) at the superficial femoral (**A,B**) and popliteal artery (**C,D**) levels in a 59 yo male with diffuse mixed atherosclerosis. VMI, virtual monoenergetic images.

**Figure 3:**
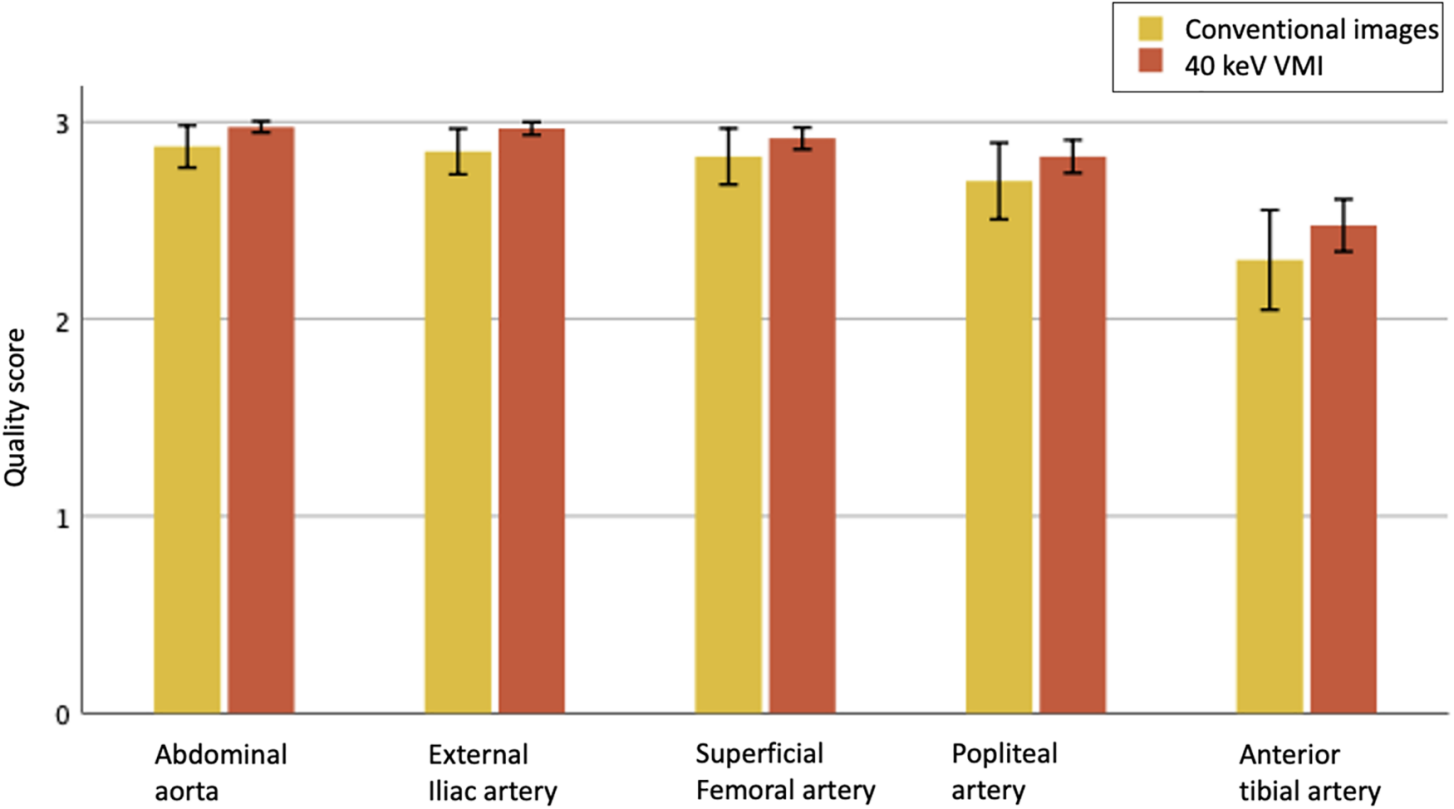
Qualitative image quality scores for evaluated vascular segments between conventional images (yellow) and 40 keV VMI (orange), on a scale of 1 to 3, displayed as means with 95% CI. Only 2 and 3 are considered of diagnostic quality.

An example of conventional images and 40 keV VMI in a patient with PAD is shown in Figure 2. The total median quality score was optimal for both modalities, with 3 [IQR, 3, 3] for conventional images vs. 3 [IQR, 3, 3] for 40 keV VMI. Proximal segments obtained better scores than distal segments. Both modalities were equal for the abdominal aorta, external iliac arteries, and superficial femoral arteries, with scores of 3 [IQR, 3, 3] vs. 3 [IQR, 3, 3]. The first differences were observed at the popliteal level, where more medium results were reported on the conventional images, with a score of 3 [IQR, 2, 3] vs. 3 [IQR, 3, 3]. Conventional images only were insufficient at the tibial level, with a score of 2 [IQR, 2, 3] vs. 3 [IQR, 2, 3] for 40 keV VMI. Qualitative scores were upgraded for 51 (26%) of patients with 9 (5%) cases upgraded from nondiagnostic to diagnostic with the use of 40 keV VMI. The most prevalent upgraded segment was the anterior tibial artery, accounting for 45% of total upgrades and 88% of nondiagnostic to diagnostic upgrades. Mann-Whitney U test showed statistically significant differences between conventional and VMI images only for the abdominal aorta and external iliac artery segments (*p* = 0.01 for both). Quality scores are provided in Table 3 and Figure 3. The inter-reader agreement was substantial (kappa = 0.62) for conventional images and moderate (kappa = 0.46) for 40 keV VMI. The most prevalent disagreement for conventional images was between the grades 2 and 3 with 19 (9.5%) disagreements. The most prevalent disagreement for 49 keV VMI images was also between the grades 2 and 3 with 29 (14.5%) disagreements.

**Table 3:** Qualitative image analysis.

Segment	Median score [IQR]			Score upgrade		Saved exams	
	Conventional images	40 keV VMI	*p* value	Conventional images	40 keV VMI	Conventional images	40 keV VMI
Abdominal aorta	3 [3, 3]	3 [3, 3]	0.01	0 (0%)	5 (8%)	0 (0%)	0 (0%)
External iliac artery	3 [3, 3]	3 [3, 3]	0.01	0 (0%)	6 (10%)	0 (0%)	0 (0%)
Superficial femoral artery	3 [3, 3]	3 [3, 3]	0.16	0 (0%)	6 (10%)	0 (0%)	0 (0%)
Popliteal artery	3 [2, 3]	3 [3, 3]	0.20	0 (0%)	12 (20%)	0 (0%)	1 (2%)
Anterior tibial artery	2 [2, 3]	3 [2, 3]	0.19	0 (0%)	22 (37%)	0 (0%)	8 (13%)
All segments	3 [3, 3]	3 [3, 3]	0.11	0 (0%)	51 (26%)	0 (0%)	9 (5%)

VMI, virtual monoenergetic images; IQR, interquartile range.

### 3.3. Quantitative image quality

A normality Kolmogorov–Smirnov test showed normal distribution of CT numbers as well as SNR and CNR measurements (*p* > 0.05), except for the distribution of popliteal artery CT numbers on conventional images (*p *= 0.023) as well as tibial artery attenuation in 40 keV VMI (*p *= 0.035).

40 keV VMI yielded higher attenuation measurements than conventional imaging, with mean values of 687 ± 259 HU vs. 303 ± 96 HU, all *p* < 0.001. SNR and CNR measurements also showed higher values for 40 keV VMI than conventional images, with mean values of respectively 12.2 ± 5.1 vs. 5.2 ± 1.8 and 28.1 ± 15.3 vs. 10.7 ± 5.2, all *p* < 0.001.

**Figure 4:**
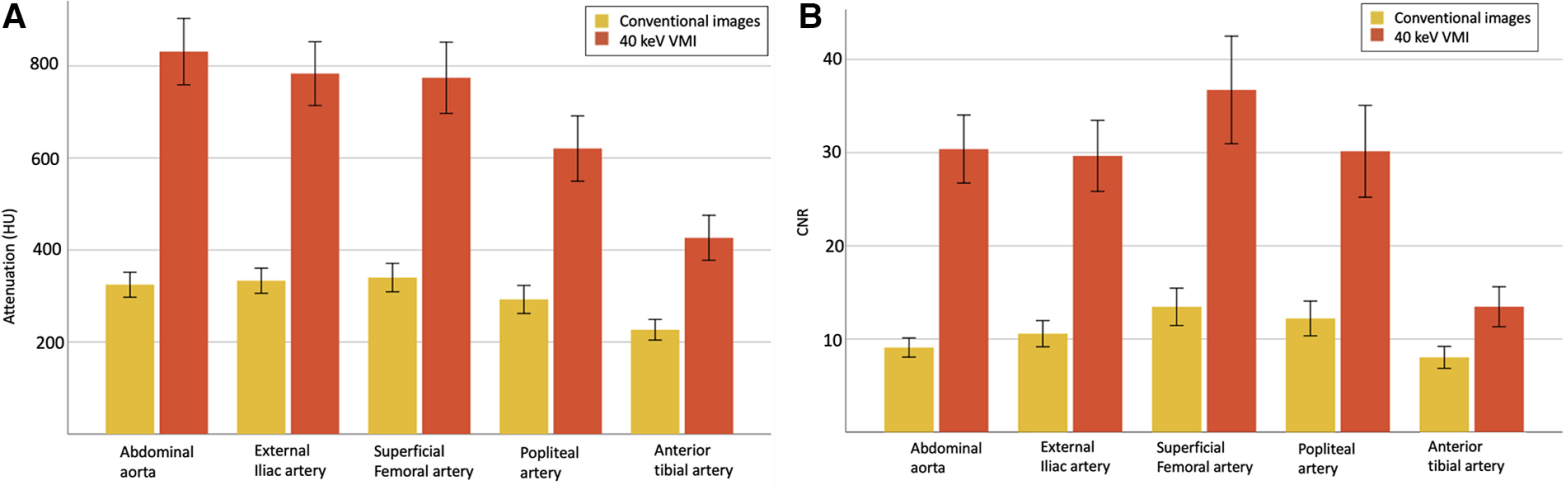
Quantitative analysis with mean attenuation (**A**) and CNR (**B**) with 95% CI, for evaluated vascular segments between conventional images (yellow) and 40 keV VMI (orange). VMI, virtual monoenergetic images; CI, confidence interval; CNR, contrast-to-noise ratio.

Of the 200 measured segments, all 200 (100%) were adequately opacified (threshold of >150 HU) on 40 keV VMI compared to 192 (96%) on conventional images, with decreasing scores in more distal segments (100% for aorta and external iliac arteries, 98% for superficial femoral and popliteal arteries and 85% for tibial arteries). Similar results were observed for CNR, in which all 200 segments (100%) achieved adequate CNRs (threshold of >3.0) on 40 keV VMI compared to 196 (98%) for conventional images (100% for aorta, external iliac and superficial femoral arteries, 98% for popliteal arteries and 93% for tibial arteries). HU and CNR values are reported in Table 4 and Figure 4.

**Table 4:** Quantitative image analysis.

Segment	Mean Attenuation (HU)			Mean CNR		
	Conventional images	40 keV VMI	*p* value	Conventional images	40 keV VMI	*p* value
Abdominal aorta	342 ± 35	831 ± 29	<0.001	9.1 ± 3.2	30.4 ± 11.4	<0.001
External iliac artery	333 ± 30	783 ± 28	<0.001	10.6 ± 4.4	29.7 ± 11.9	<0.001
Superficial femoral artery	340 ± 20	774 ± 19	<0.001	13.4 ± 6.3	36.7 ± 18.1	<0.001
Popliteal artery	293 ± 14	620 ± 15	<0.001	12.2 ± 5.8	30.1 ± 15.4	<0.001
Anterior tibial artery	227 ± 18	426 ± 32	<0.001	8.0 ± 3.7	13.5 ± 6.7	<0.001
All segments	303 ± 96	687 ± 259	<0.001	10.7 ± 5.2	28.1 ± 15.3	<0.001

VMI, virtual monoenergetic images.

## 4. Discussion

**Figure 5:**
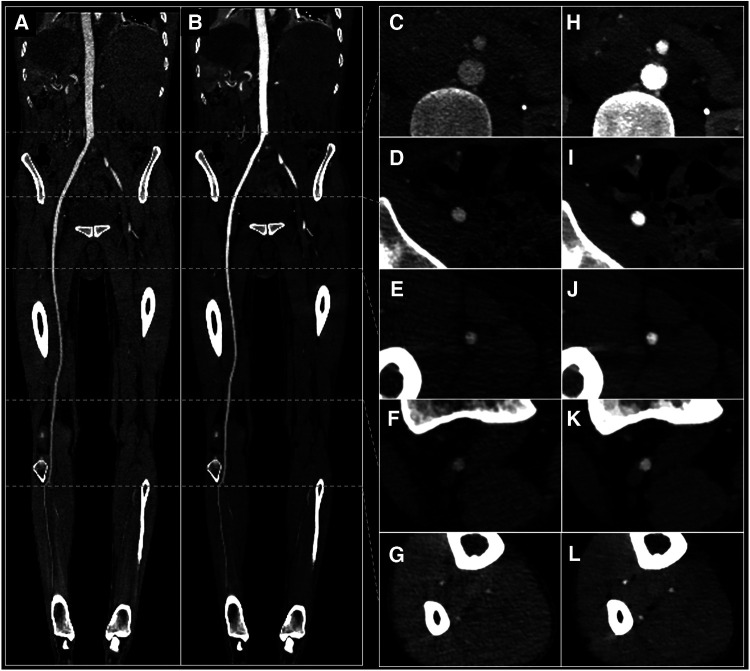
29 yo male with suspected arterial embolism. Conventional images (**A,C–G**) and 40 keV VMI (**B,H–L**) with curved MPR view (**A,B**) and axial views of the abdominal aorta (**C,H**), external iliac artery (**D,I**), superficial femoral artery (**E,J**), popliteal artery (**F,K**) and anterior tibial artery (**G,L**). VMI, virtual monoenergetic images.

Our study evaluated and demonstrated the potential of a low-contrast media volume protocol for CTA of the lower extremities using dual-energy CT and 40 keV VMI spectral imaging. Our results showed that this protocol is clinically feasible, with good qualitative and quantitative image quality scores. In another study, we already demonstrated the feasibility of such a low contrast media volume protocol using DECT for coronary CTA (30). To our knowledge, this is the only study in which a 40 ml low iodine volume is used in conjunction with the contribution of 40 keV VMI in the lower extremities. In a study including 34 patients, Almutairi and al. showed that a low contrast medium volume of 0.75 ml/kg DECT with 65 keV VMI resulted in images quality comparable to routine contrast medium volume (26). Similar results were observed by Baxa and al. with a 50 ml injection protocol (10 ml test bolus and 40 ml full bolus) in 92 consecutive patients, but with conventional CT only (19). More recently, Horehledova et al. also showed good image quality in a low volume protocol (15 ml test bolus and 30 ml main bolus) in 50 patients using conventional CT (20). While such protocols are feasible, one of the main technical challenges is acquiring the images at the right timing, as a low contrast volume protocol means a shorter bolus, thus a narrower margin of error with image acquisition. There is a significant risk of acquiring images either too soon or too late, resulting in a “diluted” aspect of the lower extremity arteries' opacification, impairing image interpretation and clinical diagnosis. Conventional image quality scores get progressively lower for the more distal arteries, particularly below the superficial femoral artery level. This drop in quality scores is partially due to contrast dilution, but also to a smaller caliber of the arteries as well as a more important impact of calcifications on the visualization of the vessel lumen. Several technological improvements can help overcome these limitations. Contrast dilution can be greatly improved with the use of spectral imaging with low virtual monoenergetic reconstructions. Indeed, we demonstrated that 40 keV VMI improved image quality compared with conventional imaging, especially on distal arteries where contrast is most likely to be diluted. At the tibial artery level, subjective quality scores went from a median value of 2 (sub-optimal) to 3 (optimal) and 22 (37%) quality scores were upgraded, including 8 (13%) from a nondiagnostic to a diagnostic score of 40 keV VMI reconstructions. Quantitative analysis showed the same improvements, with attenuation values above the quality threshold of 150 HU going from 85% to 100%, and CNR values above the quality threshold of 3 going from 93% to 100% for the tibial artery level. An example of a patient with diluted contrast media and differences between conventional and 40 keV reconstructions is shown in Figure 5. Concerning smaller artery caliber in distal segments, this is a well-known limitation that is deeply linked with CT-scan spatial resolution. Indeed, small arteries such as the anterior tibial arteries have a typical diameter of 1.5–2 mm and while their permeability can be easily assessed when the artery is normal, the same task can be very challenging or even impossible when the artery has severe atherosclerosis. While using larger matrices size such as 1,024 instead of 512 is helpful (35), current CT-scanners are limited by a spatial resolutions around 0.5 −0.625 mm (36, 37). However, the next generation of CT-scanners, i.e., spectral photon counting CT-scanners (SPCCT), are expected to greatly improve this problem thanks to their detectors that can achieve ultra-high spatial resolution up to a resolution of 200 *µ*m (38). Furthermore, arterial calcification can cause the so-called “blooming” artifacts in the artery, resulting in stenosis overestimation, which can be especially challenging and small arteries. Calcium attenuation values can also be close to those of iodine, distinguishing between both materials can sometimes be difficult when the artery is severely calcified (39). This problem is also expected to be improved with SPCCT technology, which can achieve reduced blooming artifacts and better iodine characterization (40–42). SPCCT also has the advantage of improved spectral resolution, enabling even further optimization of low-contrast media protocols (43–46). For example, in a reduced contrast media protocol of the aorta including 100 patients, Higashigaito et al. showed that 50 keV SPCCT VMI provided 25% higher CNR and non-inferior image quality than conventional CT images (47). Overall, low contrast media protocols are dependent on CT-scanner technology, patient's weight and arterial circulation, the extent of PAD, and protocol implementation. For the latter point, it is important to be aware that table speed must be adapted so that it does not outrun the bolus (faster table speed) or, on the contrary, so that the bolus does not overtake the table (slower table speed). Furthermore, radiologic technologists must be well-trained in vascular CTA protocols and aware of the right image acquisition timing.

Our study is subject to several limitations. First, we did not compare our low-contrast media volume protocol with conventional high-volume protocols, either in the same or in different patients, which could have provided more compelling results. Second, we did not assess artery stenoses, blooming artifacts, or impact of other factors such as cardiac function, which could have provided more information on the quality of the protocol. Finally, the amount of contrast media used was the same for each patient regardless of BMI or risk factors, which could be used as input variables to adapt the contrast volume for a potentially improved protocol quality.

## 5. Conclusion

Low-iodine volume dual-energy CTA of the lower extremities using 40 ml of iodinated contrast media provided images of diagnostic quality. The use of spectral 40 keV virtual monoenergetic imaging enabled a better assessment of the distal arteries with improved contrast attenuation, SNR and CNR. This protocol achieves up to 70% iodine volume reduction compared with current standard established guidelines.

## Data Availability

The raw data supporting the conclusions of this article will be made available by the authors, without undue reservation.
